# Doctors’ and nurses’ use of expectancy effects in clinical practice

**DOI:** 10.1192/j.eurpsy.2022.1769

**Published:** 2022-09-01

**Authors:** S. Vambheim, M. Bystad, J. Kirkøen, P. Aslaksen, R. Wynn

**Affiliations:** 1 UiT The Arctic University of Norway, Department Of Psychology, Tromsø, Norway; 2 University Hospital of North Norway, Department Of Geriatric Psychiatry, Tromsø, Norway; 3 UiT The Arctic University of Norway, Department Of Clinical Medicine, Tromsø, Norway

**Keywords:** Placebo, Survey, Provider-patient interaction, Treatment expectations

## Abstract

**Introduction:**

Positive treatment expectations among patients are associated with reduced symptoms and reduced negative emotions, stress and anxiety. Patient expectations may be influenced by practitioners who focus on increasing positive treatment effects and reducing psychological and physiological stress.

**Objectives:**

This study examined clinicians’ self-reported utilization of expectancy effects as additive effects to active treatments.

**Methods:**

We applied a questionnaire to investigate clinicians’ utilization of patients´ treatment expectations. The items mapped reasons for increasing patient expectations, ways through which this was done, the frequency and efficiency of increasing expectations, and the understanding of underlying mechanisms of increasing patient expectations. Nurses (N=84) and medical doctors (N=49) employed in general practitioners’ offices, hospitals, nursing homes and home health care services, responded anonymously.

**Results:**

When asked if they had tried to influence patient’s expectations to achieve an additive effect to active treatment, 71.2% reported that they had done so at least one time over the last year, 18.5% at least once per month, 16.9% at least once per week and 32.3% at a daily basis. Neither profession nor practitioner sex influenced these results. The two most frequently reported reasons for trying to influence expectations were to increase the effect of an active treatment and to calm the patient. Optimism and empathy were the two most frequently reported ways through which expectations were influenced.

**Conclusions:**

The strategy of utilizing expectation effects as additive effects to active treatment was frequent among the respondents. The main reported reasons were to increase treatment effects and reduce patients’ stress through expressing optimism and empathy.

**Disclosure:**

No significant relationships.

